# New tools for evaluating protein tyrosine sulfation and carbohydrate sulfation

**DOI:** 10.1042/BCJ20180480

**Published:** 2018-10-05

**Authors:** Sharon Yeoh, Richard Bayliss

**Affiliations:** The Astbury Centre for Structural Molecular Biology, School of Molecular and Cellular Biology, Faculty of Biological Sciences, University of Leeds, Leeds LS2 9JT, U.K.

**Keywords:** chemical probes, enzymology, sulfation

## Abstract

Sulfation is a common modification of extracelluar glycans and tyrosine residues on proteins, which is important in many signalling pathways and interactions. Existing methods for studying sulfotransferases, the enzymes that catalyse sulfation, are cumbersome and low-throughput. Recent studies published in the *Biochemical Journal* have repurposed established biochemical assays from the kinase field and applied them to the characterisation of sulfotransferases. Biochemical screening of a library of kinase inhibitors revealed that compounds that target RAF kinases may also be repurposed to inhibit sulfotransferases. Together with the available structures of sulfotransferases, these studies open the door to the development of chemical tools to probe the biological functions of these important enzymes.

Biological sulfation (also called sulfonation) is a widespread covalent chemical modification of biomolecules by the addition of a sulfonyl group (${\text{SO}}_{3}^{-}$) [1]. Whereas protein kinases use ATP as a source of bioavailable phosphate to phosphorylate their target proteins, inorganic sulfate is made available for incorporation into biomolecules in the form of PAPS (3′-phosphoadenosine 5′-phosphosulfate) through a process in which ATP is first sulfated by the ATP sulfurylase enzyme to generate adenosine 5′-phosphosulfate, which is then phosphorylated on the 3′ position of the ribose ring to generate PAPS. Sulfation is carried out by a class of enzymes called sulfotransferases. Two of the most common substrates for sulfation are saccharides intended for secretion (glycan sulfation) and the side chains of tyrosine residues on proteins (tyrosine sulfation). In humans, there are 2 tyrosyl protein sulfotransferases (TPST1 and TPST2), 4 heparan sulfotransferases (HS2ST1, HS6ST1, HS6ST2 and HS6ST3) and 15 other carbohydrate sulfotransferases (CSHT1–15). These proteins are localised to the Golgi apparatus and function in the post-translational modification of proteins destined for the outer membrane or extracellular secretion. Regulated sulfation plays a vital role in many extracellular interactions and signalling pathways.

Protein tyrosine sulfation was first described in bovine fibrinogen in 1954 [2]. It has been estimated that 7% of mammalian proteins are sulfated on tyrosine residues, although the functional consequences of these post-translational modifications have been defined in only a few cases [3]. Many chemokine receptors are tyrosine sulfated in their N-terminal regions [4]. This was first described in CCR5, a co-receptor for HIV entry through recognition of CD4, and is critical for recognition by the HIV-1 gp120 protein [5]. Sulfation also contributes to the binding of chemokine ligands, such as CCL3 and CCL4. The interactions between other chemokine receptors and their native or pathogenic ligands are also enhanced by sulfation [4]. Antibodies can also be sulfated on tyrosine residues, thus increasing the repertoire of the surface that recognises antigens through a mechanism that mimics the post-translational modification of co-receptors [6].

Of the five major classes of glycosaminoglycans, hyaluronan lacks sulfate groups, while the other four are sulfated: heparan, chondroitin, dermatan and keratan [7]. Sulfated glycans fulfil important biological functions in the extracellular matrix and on the surfaces of cells, providing structural strength and mediating signalling via specific binding interactions, and therefore the process of sulfation is carefully regulated within the Golgi network. For example, variations in the sulfation patterns of heparan sulfate influence the binding of chemokines and growth factors. Misregulation of glycan sulfation (for instance due to genetic disorders) is associated with a range of developmental syndromes, inflammation, infection and other medical conditions. For instance, a subset of HS6ST1 mutations that are implicated in idiopathic hypogonadotrophic hypogonadism (IHH), a genetic disease characterised by delayed onset of puberty, map to the catalytic domain of the enzyme and may contribute to the recognition of glycan substrate [8].

To date, unlike the well-developed protein kinase field, methods for quantifying carbohydrate and protein sulfation are low-throughput and there are few chemical tool compounds available, hindering enzymatic analysis and inhibitor studies. In a pair of groundbreaking back-to-back studies published in a recent issue of the *Biochemical Journal*, Byrne et al. have implemented two biochemical assays to measure the binding of ligands to the glycan sulfotransferase HS2ST1 (heparan sulfate 2-*O*-sulfotransferase) and protein sulfotransferases TPST1/2 (tyrosylprotein sulfotransferases 1 and 2) [9,10]. The assays used are standard in the protein kinase field and in these studies have been ingeniously adapted to apply to sulfation reactions. Differential scanning fluorimetry (DSF) and mobility shift assay (MSA) are medium-throughput assays that enable the screening of hundreds, sometimes up to thousands, of different conditions. DSF, often called thermal shift, uses the increase in fluorescence of a hydrophobic dye such as SYPRO-Orange upon binding to exposed hydrophobic core residues to quantify the thermal denaturation of a protein under varying conditions, for instance changes in buffer conditions or upon the binding of ligand molecules. The microfluidics-based MSA used by Byrne et al. utilises novel synthetic fluorescent substrates — fluorescein-tagged hexasaccharide glycans or FAM-labelled peptides derived from physiological target proteins — to carry out real-time analysis of the enzyme kinetics of HS2ST and TPST 1/2, respectively.

These methods allow the identification of factors that affect enzyme activity — for example, the activity of HS2ST is stimulated by Mg^2+^ concentrations greater than 1 mM, whereas TPST1/2 were stabilised by cofactors such as CoA. It would be interesting to compare the efficiency with which different substrates are sulfated and to extend the study to the analysis of other sulfotransferases. Here, Byrne et al. used these assays in combination to screen the Published Kinase Inhibitor Set (PKIS) of 367 well-characterised kinase inhibitors against TPST1 and HS2ST. Interestingly, the ‘hit rates’ against the two enzymes were markedly different: screening at 40 µM inhibitor concentration, only three compounds inhibited HS2ST activity by greater than 50%, while more than 30 of the compounds inhibited TPST1 to a similar extent. Furthermore, all three of the top hits against HS2ST were oxindole-derived RAF kinase inhibitors that were among the top 10 hits against TPST1. In addition, rottlerin, a natural product that has activity against a broad set of biological targets including kinases, was found to inhibit both TPST1 and HS2ST.

Crystal structures of sulfotransferases include the human TPST1 in complex with adenosine 3′–5′ diphosphate (PAP) and two different substrate peptides (PDB codes 5WRI and 5WRJ); and structures of chicken HS2ST (PDB code 4NDZ) or zebrafish 6-*O*-sulfotransferase (6-OST) (PDB codes: 5T03, 5T05 and 5T0A) in complex with PAP and oligosaccharides [8,11,12]. The catalytic domain of sulfotransferases is unrelated to that of protein kinases, and moreover, the way in which the substrates are held are different. Protein kinases have a deep cleft into which ATP binds and the protein substrate binds to the surface proximal to this cleft; ATP is held in an orientation that buries the adenine base. In contrast, the active site of sulfotransferase comprises a tunnel with the binding sites for PAPS and peptide/glycan substrate is at either end; PAPS binds to the active site of sulfotransferases in an orientation that exposes the adenine base to solvent. The availability of these structures enables the binding sites of inhibitors to be predicted using computational approaches. Whereas the kinase inhibitors compete with ATP binding to RAF, docking analysis carried out by Byrne et al. suggests that these compounds do not have an equivalent mode of action in sulfotransferases: they do not simply compete with the substrate molecule PAPS. Instead, some of the compounds are predicted to bind across the peptide/glycan and PAPS-binding sites. Intriguingly, rotterlin is predicted to dock to the PAPS-binding site of HS2ST and the peptide-binding site of TPST1.

These recent studies have identified several compounds that are potentially useful for studying sulfotransferases, with the caution that they are not selective and they also inhibit kinases. Because the size and shape of the active sites are different from protein kinases, it should be possible to develop more specific and selective chemical inhibitors of sulfotransferases. Robust assays such as DSF and MSA will facilitate the determination of structure–activity relationships of inhibitors. The hit compounds from the PKIS library could be used as starting points for further chemistry, although crystal structures of the sulfotransferases in complex with the inhibitors would be helpful in confirming the predicted binding modes and guiding further chemistry. In addition, the approaches that Byrne et al. have developed could be used to screen further libraries of compounds. This need not be restricted to kinase inhibitors, and *in silico* approaches may help to narrow down the search for new hit matter. Screening simple, low molecular mass compounds (<300 Da) called ‘fragments’ using biophysical methods such as NMR spectroscopy might also be considered because this is an efficient way to scan a diverse set of molecules, with library sizes typically of 1000–2000, and can identify ‘hot-spots’ within the active site [13]. From these weakly binding fragments, potent inhibitors can be developed, such as the clinically approved BRAF inhibitor vemurafenib. With the right combination of approaches, and perhaps a little luck, it seems likely that a toolkit of inhibitors to probe the biological functions of sulfotransferanses is just around the corner.

## Abbreviations

CSHT: carbohydrate sulfotransferase
DSF: differential scanning fluorimetry
HS2ST: heparan sulfate 2-O-sulfotransferase
MSA: mobility shift assay
PAP: adenosine 3′–5′ diphosphate
PAPS: 3′-phosphoadenosine 5′-phosphosulfate
PKIS: Published Kinase Inhibitor Set
TPST: tyrosyl protein sulfotransferase
